# Patient Satisfaction and Tear Film Break-Up Time After First-Time Wearing of Silicone Hydrogel Contact Lenses

**DOI:** 10.7759/cureus.52516

**Published:** 2024-01-18

**Authors:** Naoko Misu, Tatsuya Mimura, Hidetaka Noma, Koichiro Shinbo

**Affiliations:** 1 Ophthalmology, Teikyo University School of Medicine, Tokyo, JPN; 2 Ophthalmology, Hachioji Medical Center, Tokyo Medical University, Hachioji, JPN; 3 Ophthalmology, Nerima Station West Eye Clinic, Tokyo, JPN

**Keywords:** non-invasive tear break-up time, verofilcon a, silicone hydrogel, glasses, first-time wearers, daily disposable soft contact lens

## Abstract

Purpose: The dryness and discomfort associated with soft contact lenses (SCLs) prevent their continued use. Recently, verofilcon A, a new daily disposable silicone hydrogel material SCL, was introduced, which has a high water content (surface water content of 80% or more) that overcomes the low water content drawback of silicone hydrogels. In this study, we evaluated the non-invasive tear break-up time (NIBUT) and comfort level in individuals wearing verofilcon A SCL for the first time.

Methods: We enrolled 42 first-time SCL wearers, comprising 84 eyes. NIBUT was measured using the DR-1α® dry eye observation device at the state of the naked eye before SCL wear (baseline) and at one and four weeks after the first SCL wear. Additionally, we conducted a questionnaire survey during the fourth week to assess the comfort level (0-10) of SCL wear.

Results*:* The NIBUT values were significantly higher at one week (10.8 ± 2.2 s, p<0.01) and four weeks (11.4 ± 2.2 s, p<0.01) after the first SCL wear than those at baseline (5.9 ± 2.0 s). Comfort level in SCL use was significantly higher at one week (9.0 ± 1.1, p<0.01) and four weeks (8.7 ± 1.2, p<0.01) than that at baseline (7.8 ± 1.8), and this level was higher regardless of the baseline NIBUT value.

Conclusion: Prolonged BUT and increased comfort levels were observed in individuals wearing verofilcon A SCLs. Improvement in tear fluid retention was found to alleviate dry eye and discomfort, suggesting that verofilcon A may be a beneficial introductory lens for first-time SCL wearers.

## Introduction

Dry eyes and ocular discomfort are the main reasons for discontinuing soft contact lens (SCL) wear [1]. Previous reports observed a dropout rate of approximately 40% among CL wearers abandoning their CLs within four months [2]. In addition, a literature review of studies conducted over the past 20 years reported that 23%-94% of CL wearers experienced discomfort [3]. Furthermore, studies have reported that approximately 12%-51% of CL wearers discontinue using CLs [2,4-7]. These studies highlight the importance of selecting comfortable SCLs that are less likely to cause dry eye symptoms in first-time SCL wearers to avoid discontinuation of SCL wear.

In Japan, a new silicone hydrogel SCL (SiHy-SCL) (Precision 1®, Alcon Japan, Inc., Tokyo, Japan) made of verofilcon A material was introduced in March 2021. Verofilcon A was first introduced in Oceania and the United States, and comfort studies on this material have been conducted in Australia/New Zealand and the United States [8,9]. A survey conducted in Australia and New Zealand involving 129 new SCL wearers aged 18 years and older revealed that 91% agreed that "verofilcon A is the preferred lens," 79% agreed that "verofilcon A gives me the option of not wearing glasses," and 70% agreed that " verofilcon A is the best lens to start the contact lens wearing experience" [8]. In the United States, a randomized crossover clinical trial was conducted with two different SCLs, verofilcon A and etafilcon A, in 96 participants who were already wearing CL [9]. The results demonstrated that 73.9% of the participants preferred verofilcon A to etafilcon A. Additionally, satisfaction with visual acuity, SCL handling, and wearing comfort was significantly better for verofilcon A than for etafilcon A [9]. These results suggest that verofilcon A provides a positive wearing experience among SCL users.

Decreased ocular surface moisture is the most important factor preventing continued SCL wear. The SCL placed on the corneal surface divides the tear film into two interfaces: the pre-SCL and post-SCL tear films [10,11]. Of these, the amount of lipid layer and aqueous phase in the former pre-SCL tear film depends on the contact lens material and design [10,12]. To date, various non-invasive methods have been employed for measuring tear film break-up time (BUT) on the SCL surface to assess non-invasive BUT (NIBUT), including (1) direct observation of the SCL surface [13], (2) observation of the pre-SCL tear film [14], (3) assessment of color patterns using lipid layer interferometry [15,16], (4) measurement of tear fluid meniscus height [17], and (5) the use of the tear interferometer DR-1α® [18]. Among these, the DR1-α is well-established as an instrument that can measure NIBUT objectively and reproducibly. Therefore, in this study, the DR-1α® was used to measure the tear fluid breakup time on the CL surface.

Verofilcon A was developed as an SCL with a SiHy-SCL water gradient. The lens surface consists of a SiHy core containing 33% water, while the outer surface forms a layer containing 80% water [19]. The SCL has a lubricated surface with continuous high oxygen transmission [19]. This study aimed to measure NIBUT on the verofilcon A material SCL surface in first-time SCL wearers and examine the relationship between NIBUT and comfort experienced with verofilcon A SCL wear.

This article was previously posted to the medRxiv preprint server on June 29, 2023.

## Materials and methods

Research design

This was an investigator-initiated, observational, prospective cohort study conducted in accordance with the ethical guidelines of the Declaration of Helsinki (World Medical Association 2013) and the Ethical Guidelines for Medical Health. The studies involving human participants were approved by the Teikyo University Ethical Review Committee (#19-211, #20-166). The study, along with several related studies, was registered as a clinical trial in the University Medical Information Network for Clinical Trials (UMIN-CTR) (UMIN registration numbers: UMIN000041107 and UMIN000042265). This study was conducted between April 2021 and May 2022 at the outpatient clinic of the Nerima Station West Eye Clinic and Department of Ophthalmology, Teikyo University School of Medicine. Written informed consent was obtained from all the participants after a complete explanation of the study content.

Participants

The inclusion criteria were as follows: (1) healthy participants; (2) age ≥ 12 years; (3) used glasses; (4) no history of CL wear; (5) myopic astigmatism; (6) refraction of −0.5 to −6.0 D; and (7) best corrected visual acuity of 20/25 or better. The exclusion criteria were as follows: (1) age < 12 years; (2) ocular or systemic disease; (3) history of refractive surgery; and (4) corneal epithelial erosion. During the study, participants were allowed to use artificial tear drops with the CLs. Participants were selected from among patients who wanted to wear CLs for the first time. Forty-two participants were included in the study and tested for tear film stability. The age ranged from 12 to 63 (mean ± deviation, 18.4 ± 9.5) years. A total of 18 men and 24 women participated in the study.

Study schedule

The NIBUT was measured using a tear interferometry DR-1α® (Kowa Co., Nagoya, Japan). It was measured on the corneal surface of the eye at baseline and on the SCL surface with SCL worn at one and four weeks after SCL wear. NIBUT measurements were obtained for both eyes, and the average value was calculated.

Participants were asked to complete questionnaires regarding their experiences with wearing eyeglasses and SCLs at three time points: before the first SCL wear and one and four weeks after the first SCL wear. Registered participants underwent lens fitting by a CL specialist with more than 10 years of practical experience. Participants also received one hour of training and practice by a CL practitioner to insert and remove SCLs by themselves. Participants were instructed to wear the SCLs after they were able to wear them properly. They were instructed to wear SCLs for 8-10 hours per day for five days a week and glasses on other days of the week. The participants were informed that the SCLs were intended for daily disposable use and should be discarded after each day of wear. Examinations were conducted at baseline and after one and four weeks of the first SCL wear. All 42 participants enrolled in the study completed the one-month follow-up period.

Characteristics of the evaluated SCLs

Verofilcon A (PRECISION 1®) daily disposable SCLs composed of SiHy were used. Verofilcon A is made from a new high oxygen permeability (Dk; 90 × 10−11 barriers) material with a 2-3 µm thick surface, containing more than 80% water content, and is a class 1 ultraviolet blocker (≥ 90% of UVA, ≥ 99% of UVB) [19]. Verofilcon A SCLs have a smooth surface owing to SMARTSURFACE® technology.

Grade of corneal superficial punctate keratopathy and bulbar conjunctival hyperemia

During the initial visit to our clinic, ocular findings were assessed through a slit-lamp examination. Objective changes in corneal superficial punctate keratopathy (SPK) and bulbar conjunctival hyperemia were classified into four grades (0 = normal, 1+ = mild, 2+ = moderate, or 3+ = severe) [20,21]. Specifically, the corneal epithelial erosion score was determined using fluorescein staining. The following scores were used: 0 = none, 1+ = < 1/3 of the total corneal area, 2+ = between 1/3 and 2/3 of the total corneal area, and 3 + = > 2/3 of the total corneal area. The following scores were used for conjunctival hyperemia: 0 = no hyperemia (-) normal findings, 1+ = mild (+) few dilated blood vessels, 2+ = moderate (++) many dilated vessels, and 3+ = severe (++) dilated vessels.

Questionnaires

The questionnaire consisted of four parts: 1) a questionnaire assessing the comfort level of wearing glasses at baseline, and 2) three questionnaires evaluating the comfort and impression of wearing SCLs. We used the modified questionnaire scores by Marx et al. for assessment [8,22].

The first questionnaire consisted of one item assessing the comfort of wearing glasses and SCLs. Participants were rated on a scale of 1 (poor) to 10 (excellent) depending on whether they were comfortable while wearing glasses or SCLs overall (throughout the day) [8,22]. Questionnaires were administered before wearing the SCLs (at the baseline visit) and at one and four weeks after the first SCL wear.

The second questionnaire consisted of three items related to participants’ impressions of wearing SCLs, as follows: Q1 = More comfortable than expected, Q2 = As comfortable as no lenses at all. Q3 = So comfortable, I barely felt anything [8, 22]. The participants were asked to indicate whether they agreed (yes) or disagreed (no) with each item, and the questionnaire was administered four weeks after the SCLs were fitted. Based on the scores for each item in the questionnaire, the severity of ocular symptoms and participants’ satisfaction with SCL comfort and vision were analyzed.

Statistical analyses

The two-tailed paired Student’s t-tests were used to determine the significance of the differences between the two groups. Correlations among the variables were determined by calculating two-tailed Pearson correlation coefficients. Data were expressed as means ± standard deviation or percentages. Statistical analyses were performed using the SAS System software version 9.1 (SAS Institute Inc., Cary, NC, USA), and the level of significance was set at p<0.05.

## Results

Participant characteristics

A total of 42 participants were enrolled, and all of them completed the study without any dropouts. The right and left eyes of participants had similar values for mean refractive error (−3.9 ± 1.6 vs. −3.8 ± 1.5 D), sphere (−3.5 ± 1.6 vs. −3.4 ± 1.4 D), and cylinder (−0.7 ± 0.4 vs. −0.8 ± 0.4 D). The refractive power of the SCLs used by the participants was (−2.9 ± 1.4 D) and (−2.8 ± 1.4 D) in the right and left eyes, respectively.

Changes in NIBUT over time

**Figure 1:**
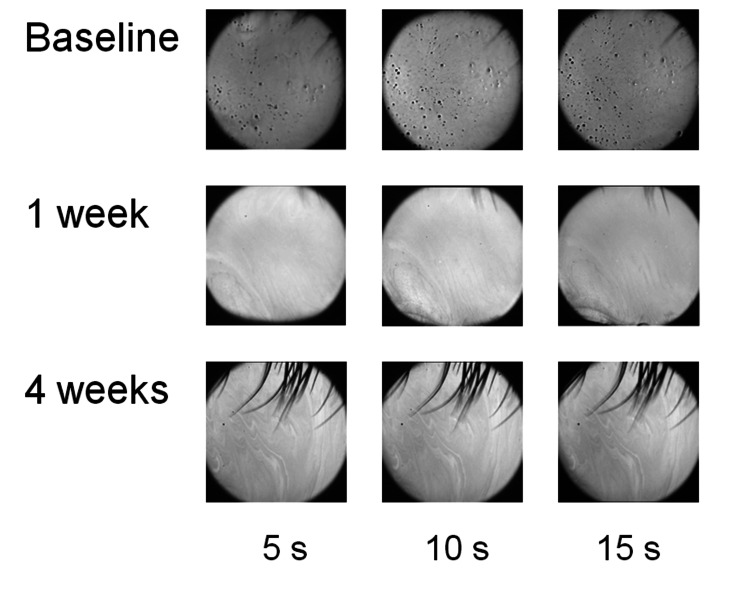
Typical non-invasive tear break-up time (NIBUT) measurements (right eye of a 19-year-old boy). Top: Baseline. Middle: one week after the first soft contact lens (SCL) wear. Lower: four weeks after the first SCL wear.

**Figure 2:**
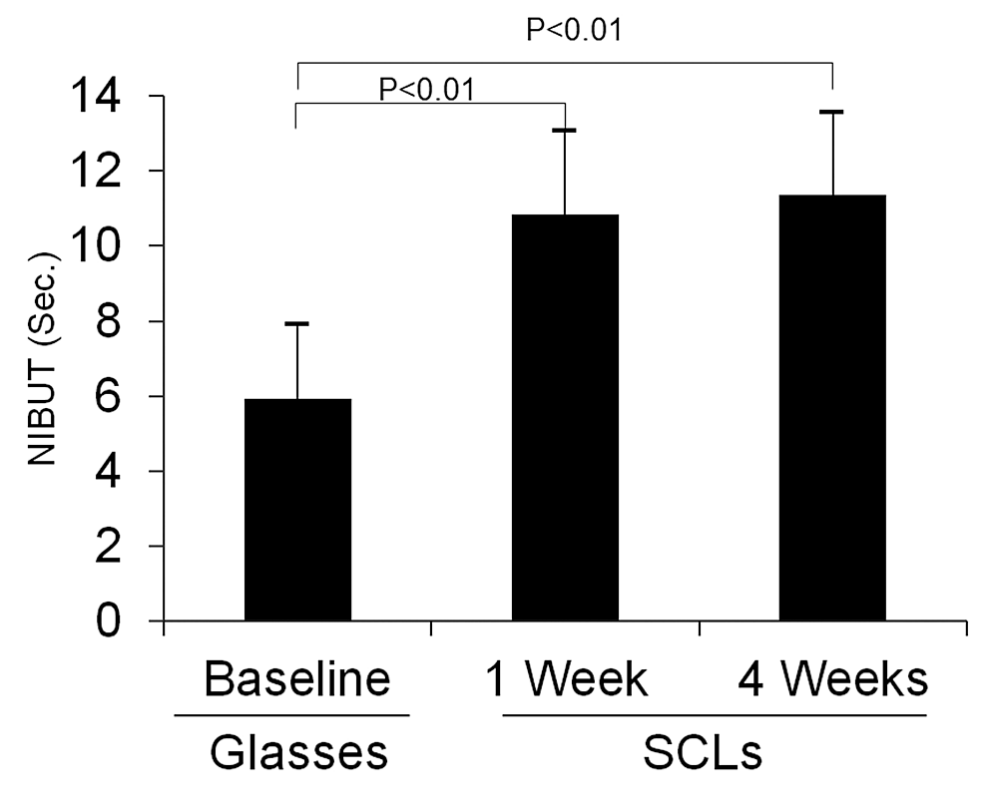
Time course of non-invasive tear break-up time (NIBUT) (n=42). NIBUT is averaged over both eyes and compared at baseline, one week, and four weeks after the first soft contact lens (SCL) wear. A two-tailed paired student's t-test is used for comparison between the two groups.

**Figure 3:**
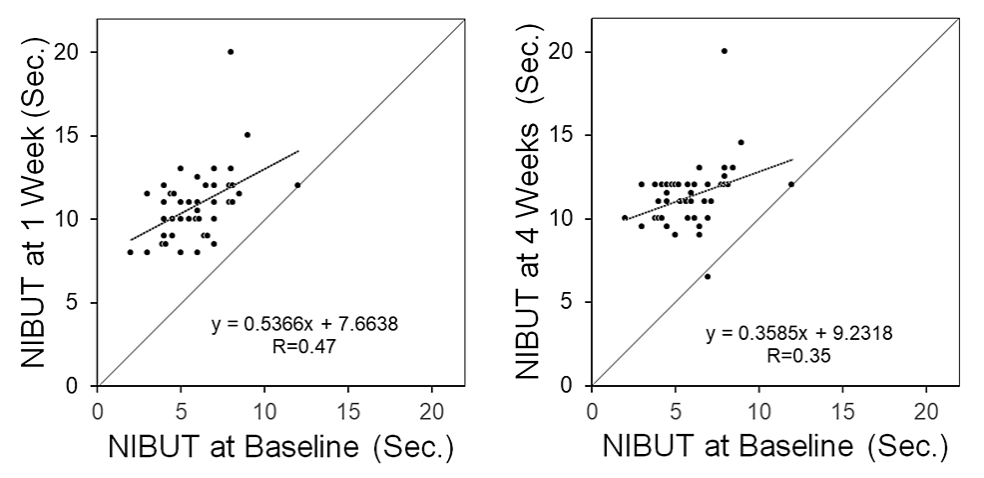
Correlations between non-invasive tear break-up time (NIBUT) at one and four weeks after the first soft contact lens (SCL) use and baseline NIBUT (n=42). Correlations among the variables are determined by calculating two-tailed Pearson correlation coefficients.

Figure 1 shows a typical ocular surface tear layer observed with DR1-α® (right eye of a 19-year-old boy). At baseline, proteinaceous deposits in the tear fluid and the breakup of the tear fluid layer were observed 5 s after eyelid opening. In the first week after the first SCL wear, a dry spot appeared at 7 o'clock for 10 s, while in the fourth week, no tear film break-up was observed until 20 s. The mean NIBUT values for 42 eyes were calculated as follows: 5.9 ± 1.9 s (baseline, without SCL), 10.8 ± 2.2 s (one week with SCL), and 11.4 ± 1.9 s (four week with SCL), showing that the NIBUT was significantly longer at one week (p<0.01) and four weeks with SCL (p<0.01) compared to that at baseline (Figure 2). Figure 3 shows a significant correlation between the NIBUT at one and four weeks of SCL wear relative to the NIBUT at baseline (r=0.47, p<0.01; r=0.35, p=0.02).

Evaluation of the ocular surface using slit-lamp microscopy

**Figure 4:**
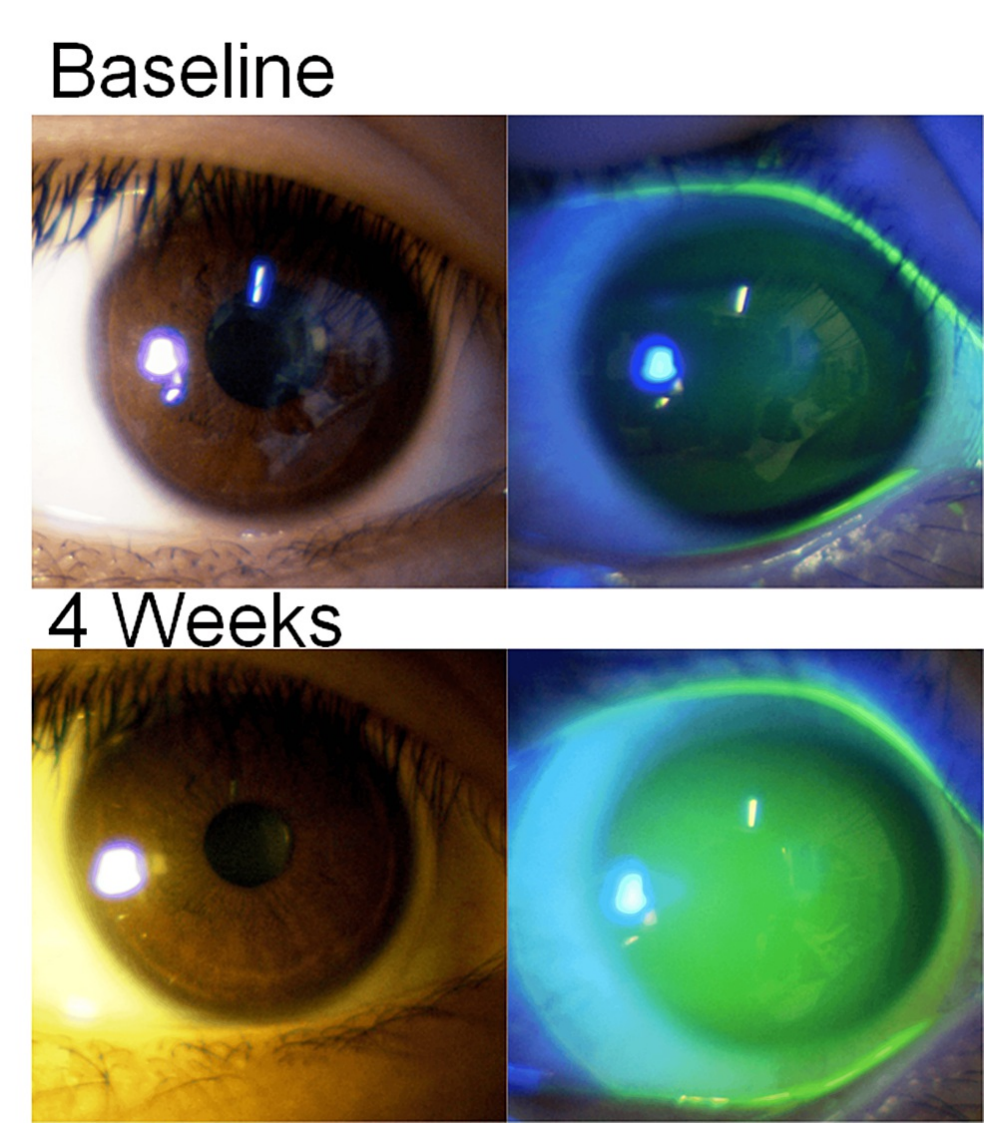
Typical slit-lamp micrographs of the ocular surface before (baseline) and at four weeks of soft contact lens (SCL) wear (right eye of a 17-year-old boy).

**Figure 5:**
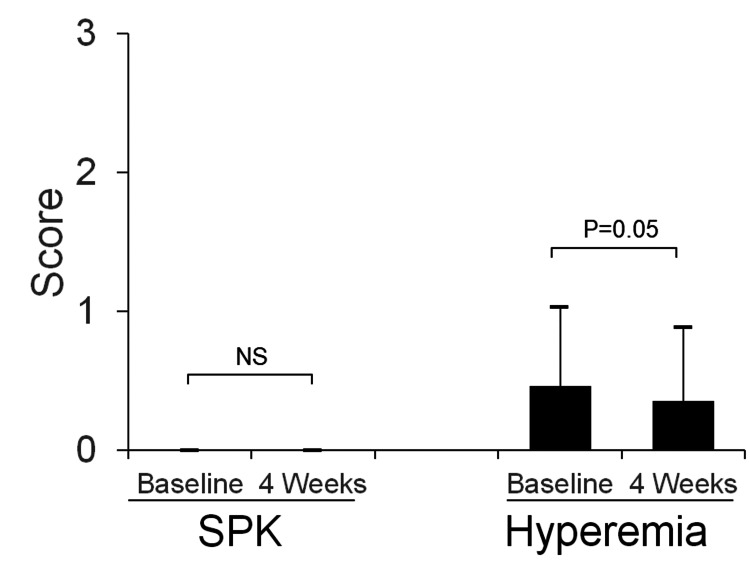
Scoring of superficial punctate keratitis (SPK) and conjunctival hyperemia (0–3) (n= 84 eyes from 42 patients). Scores are compared before soft contact lens (SCL) wear (baseline) and at four weeks. The two-tailed paired Student's t-test is used for comparison between the two groups. NS: Not significant.

Figure 4 shows a typical photograph of the ocular surface (right eye) of a 17-year-old boy. This patient had no corneal erosions at baseline or at week four after SCL fitting, and the SCL fitting was good in both eyes. The SPK and hyperemia scores at baseline and four weeks (N=84 eyes) are shown in Figure 5. SPK was absent in all patients at both baseline and four weeks, and both scores were 0 ± 0. The conjunctival hyperemia score was 0.5 ± 0.7 at baseline and 0.5 ± 0.7 at four weeks with no deterioration.

Evaluation of visual comfort level

**Figure 6:**
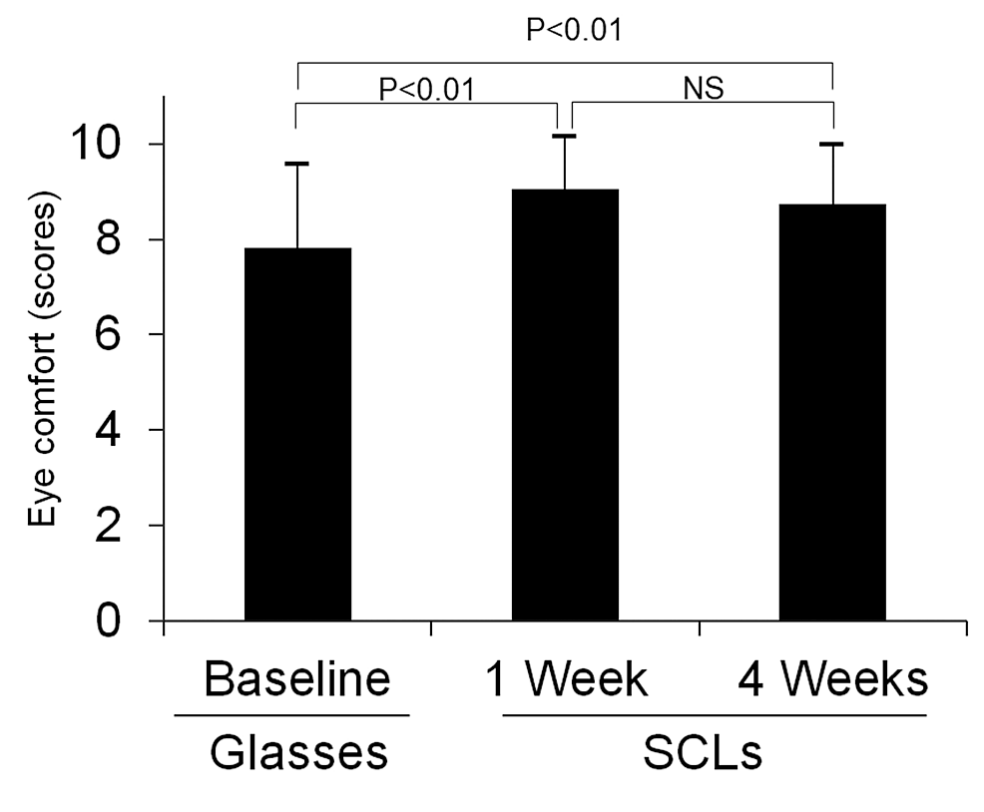
Mean eye comfort scores throughout the day (0–10), with scores compared before soft contact lens (SCL) wear (baseline) and at one and four weeks of SCL wear (n=42). The two-tailed paired Student's t-test is used for comparison between the two groups. NS=Not significant.

The comfort levels of the 42 patients at baseline (with glasses) and at one and four weeks of SCL wear are shown in Figure 6. Comfort level was significantly higher at one week (9.0 ± 1.1, p<0.01) and four weeks (8.7 ± 1.2, p<0.01) than that at baseline (7.8 ± 1.8).

**Figure 7:**
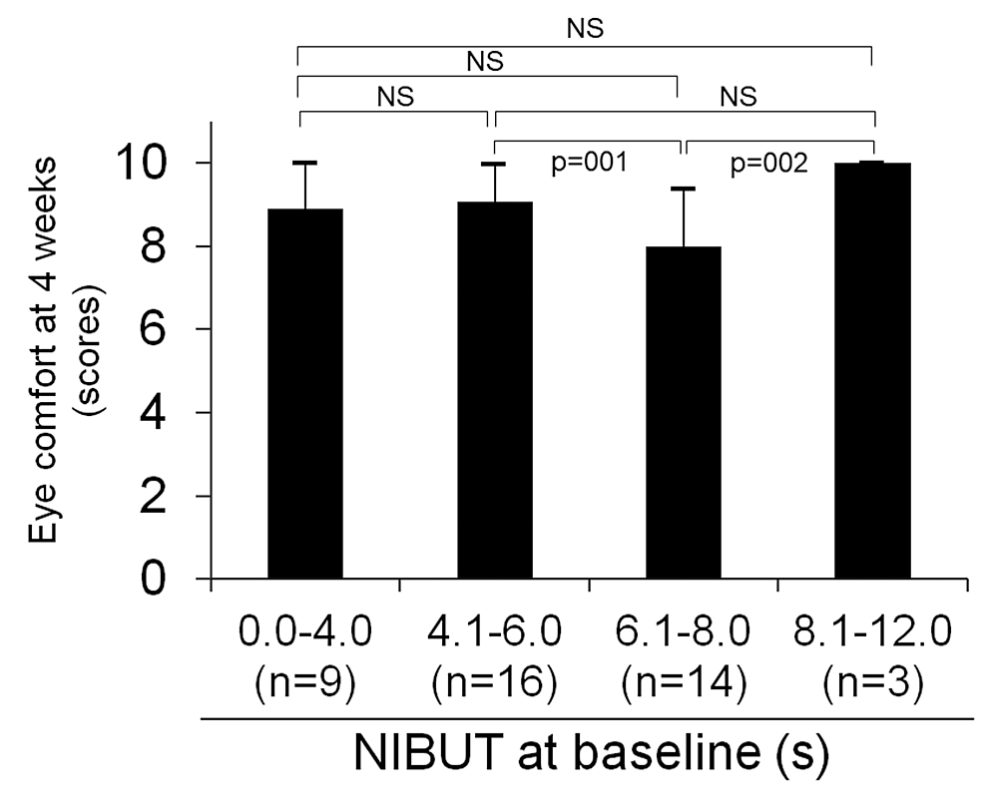
Comparison of eye comfort scores at four weeks after the first soft contact lens (SCL) wear between four groups based on the amount of non-invasive tear break-up time (NIBUT) at baseline (n=42). NIBUT: mean of both eyes. *Two-tailed paired Student's t-tests, NS: Not significant.

The participants were then divided into four groups based on the mean NIBUT values of both eyes at baseline as follows: BUT (0.0-4.0 s) (n=9), (4.1-6.0 s) (n=16), (6.1-8.0 s) (n=14), and (8.1-12.0 s) (n=3). Comparison of comfort level at four weeks of SCL wear among these four groups revealed no significant difference between the BUT (0.0-4.0) group, with the lowest value, and the other three groups. The comfort scores at four weeks were as follows: BUT (0.0-4.0 s): 8.9 ± 1.1, (4.1-6.0 s): 9.1 ± 0.9, (6.1-8.0 s): 8.0 ± 1.4, and (8.1-12.0 s): 10.0 ± 0.0 (Figure 7).

Relationship between NIBUT at baseline and satisfaction level

**Figure 8:**
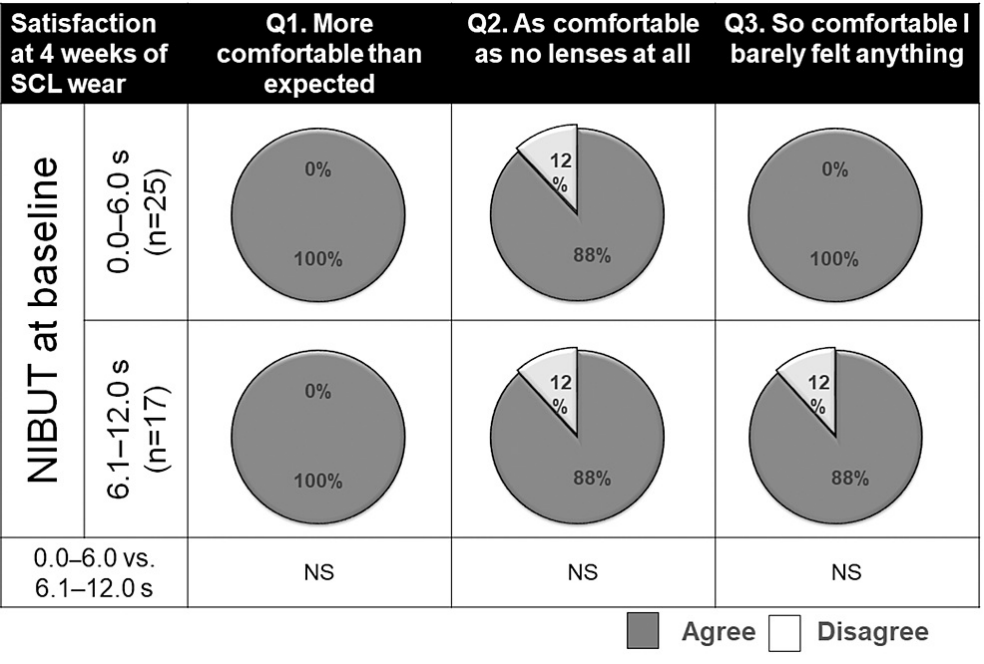
Comparison of satisfaction rates with soft contact lens (SCL) wear between the two groups divided by baseline non-invasive tear break-up time (NIBUT). The patients are divided into two groups based on the mean value of NIBUT in both eyes at baseline: (0.0–6.0 s) (n=25) and (6.1–12.0 s) (n=17). The satisfaction rates for SCL wear are compared between the two groups. *NS: not significant (the chi-square test of independence or Fisher's exact probability test).

Participants were divided into two groups based on the mean value of NIBUT in both eyes at baseline: BUT (0.0-6.0 s) (n=25) and (6.1-12.0) (n=17). The satisfaction with SCL wear was compared between the two groups, and both groups demonstrated higher satisfaction in all aspects of SCL wear. No significant differences were observed between the two groups in any of the following items: Q1: More comfortable than expected (100.0% vs. 100.0%), Q2: As comfortable as no lenses (88.0% vs. 88.0%), and Q3: So comfortable, I barely felt anything (100.0% vs. 88.0%) (Figure 8).

## Discussion

Summary of the results

Our results demonstrated a significant increase in the mean NIBUT with verofilcon A at one and four weeks after the first SCL wear compared to the eye condition before SCL wear. No difference was observed in visual comfort and satisfaction between individuals with low and normal NIBUT at baseline after wearing verofilcon A SCL. These findings suggested that verofilcon A prolongs the NIBUT on the surface of SCL in first-time SCL wearers and that patients exhibit high satisfaction with the wearing experience, regardless of NIBUT duration. This suggests that verofilcon A is an effective introductory lens for first-time SCL wearers.

Comparison with other reports on NIBUT after wearing verofilcon A

In our study, we observed that wearing verofilcon A SCL resulted in a prolonged NIBUT on the SCL surface than that with the naked eye. The increased wetting of the ocular surface contributes to the satisfaction experienced by first-time SCL wearers. Previous clinical reports demonstrated a higher NIBUT on the SCL surface after 12 hours of verofilcon A SCL wear than that of senofilcon A and nesofilcon A (9.15 ± 3.08 vs. 6.82 ± 2.69 vs. 4.73 ± 1.68 s) SCL wear [23]. Additionally, in an in vitro study on SCL water wettability, verofilcon A demonstrated better water stability than that of senofilcon A [23-25]. The contact angle for assessing the wettability of the SCL surface was 41.0° for verofilcon A, which was better than that for senofilcon A (96.5°) [23,24]. In a recent clinical study, 81% of 172 SCL wearers expressed a preference for verofilcon A over other SCLs [8]. Additionally, in this study, participants reported that verofilcon A was more comfortable throughout the day, could be worn longer, and had fewer interruptions than SCLs containing senofilcon A and nesofilcon A [8]. Thus, wearing verofilcon A may have improved the water wettability of the ocular surface, leading to better comfort.

Comparison with other reports on NIBUT after wearing SCLs with water gradient technology

Verofilcon A, as well as other SCLs with SMARTSURFACE® Technology, such as delefilcon A (DAILIES TOTAL 1®, Alcon Laboratories, Inc., Fort Worth, TX) and lehfilcon A (TOTAL30®, Alcon Laboratories, Inc.), have been studied regarding their impact on NIBUT after SCL wear. In a study comparing NIBUT before and after wearing lehfilcon A SCL, mean NIBUT increased to 21.27 ± 11.97 s after SCL wear compared to 15.19 ± 9.54 s before SCL wear [11]. In another study, mean NIBUT before and after wearing Lehfilcon A SCL was compared in participants with low and high scores on the Contact Lens Dry Eye Questionnaire-8 (CLDEQ-8) [26]. In participants with low CLDEQ-8 scores (Low CLDEQ-8 group), NIBUT was prolonged from 17.92 ± 10.88 s before SCL wear to 29.77 ± 16.47 s at one month after SCL wear. In contrast, in subjects with high CLDEQ-8 scores (High CEDEQ-8 group), NIBUT was prolonged from 17.92 ± 10.88 s before SCL wear to 18.52 ± 12.91 s at one month after SCL wear [26]. In a similar study, Fujimoto et al. retrospectively compared NIBUT in 50 delefilcon A or narafilcon A SCL users. The results demonstrated that delefilcon A with the water gradient technology had a longer NIBUT than narafilcon A (4.1 ± 2.4 vs. 2.7 ± 1.6 s) [18]. In another study, the mean NIBUT of participants who routinely wore delefilcon A SCL was longer (9.2 s) than those who routinely used stenfilcon A (6.3 s) or narafilcon A (5.1 s) SCLs [14]. The results of these previous studies and ours suggested that both water gradient and SMARTSURFACE® technologies prolong NIBUT.

Relationship between SCL discontinuation and dry eye

Dry eyes and discomfort experienced while wearing SCL may lead to the discontinuation of its use. In the present study, all 42 participants were able to use verofilcon A SCLs comfortably and without failure during the one-month study period. Surprisingly, in an online survey conducted in Canada involving 4,207 SCL wearers, 40% of participants discontinued wearing SCL within four months [7]. Compared with participants who discontinued SCL, those who did not discontinue SCL had higher usage of SiHy SCLs (49% vs. 38%). The primary reason reported for the discontinuation includes discomfort (24%), followed by dryness (20%), hyperemia (7%), and cost (7%) [7].

In another study, 110 participants (aged 13-19 years) who had not previously worn SCLs were randomly assigned to nelfilcon A SCLs (DAILIES AquaComfort Plus, Alcon Research Ltd., Fort Worth, Texas) or glasses for six months. By the end of the sixth month, 13 participants had discontinued the study, including 10 (17.5%) in the SCL group and three (5.7%) in the eyeglasses group. The proportion of participants who discontinued treatment was significantly higher in the SCL group than those in the eyeglasses group (p=0.04) [27]. In a UK study involving 531 participants, the discontinuation rate of SCLs within the first year of wear was 20%-25% [28]. The most common reasons for the discontinuation of SCLs were vision problems in 41%, discomfort in 36%, and handling problems with SCLs in 25% [28]. In a clinical study of verofilcon A wear involving 70 patients (140 eyes), 138 of the 140 eyes completed the three-month study, with an interruption rate of 1.4% (one patient, two eyes). The reason for this interruption was a change in residence. Of the 138 eyes that completed the study, 136 (98.6%) maintained a visual acuity of 20/20 or better while habitually wearing verofilcon A SCL, without any interruptions due to SCL failure [29].

In our study, none of the patients discontinued wearing verofilcon A SCL. This observation had several reasons. The first reason was the short duration of our study (one month). Second, the ophthalmologist and practitioner carefully explained the SCL fitting process to the participants before initiating the fitting. Finally, the high comfort level experienced with verofilcon A SCL wear resulted in fewer dropouts. As shown in Figure 7, no difference was observed in the comfort level of wearing verofilcon A SCL between participants with a short NIBUT of 0-4 s and those with a long NIBUT of 8.1-12.0 s at baseline. In addition, as shown in Figure 8, all participants reported that wearing verofilcon A was more comfortable than they had expected, regardless of a low or high NIBUT at baseline.

Study limitations

This study had several limitations. First, this was an observational study of one group and not a randomized two-group study. Therefore, single- or double-masked groups were not used in this study. A one-group before-and-after comparative study may involve subjective bias among the study participants. Second, the study period was one month, and future long-term studies are warranted on the use of verofilcon A SCL. Third, most study participants were young because they were first-time SCL wearers. Future studies should be conducted with a wider range of age groups. Fourth, NIBUT was measured using the instrument DR1-α🄬 in this study. Comparison with other tests to measure NIBUT, such as actual observation of NIBUT under a slit-lamp microscope using fluorescein, would be necessary.

## Conclusions

In conclusion, we observed that NIBUT was prolonged after wearing verofilcon A SCL. Improved tear film instability on the surface of the SCL reduced dry eye symptoms and discomfort in SCL wearers, leading to fewer interruptions and continued use of the SCL. Verofilcon A may be useful as an introductory lens for first-time SCL users.
